# The Synergistic Effect of Functional Status and Comorbidity Burden on Mortality: A 16-Year Survival Analysis

**DOI:** 10.1371/journal.pone.0106248

**Published:** 2014-08-29

**Authors:** Cynthia Chen, Isaac Sia, Hon-ming Ma, Bee Choo Tai, Angela Cheong, Ngan Phoon Fong, Shi Yu Julia Tan, Kin Ming Chan, Boon Yeow Tan, Edward Menon, Chye Hua Ee, Kok Keng Lee, Yee Sien Ng, Yik Ying Teo, Stefan Ma, Derrick Heng, Gerald Choon-Huat Koh

**Affiliations:** 1 Saw Swee Hock School of Public Health, National University of Singapore, National University Health System, Singapore, Singapore; 2 College of Public Health and Health Professions, University of Florida, Gainesville, Florida, United States of America; 3 Departments of Medicine and Therapeutics, Prince of Wales Hospital, The Chinese University of Hong Kong, Hong Kong SAR, China; 4 Yong Loo Lin School of Medicine, National University of Singapore, National University Health System, Singapore, Singapore; 5 Medical Services, Ang Mo Kio Thye Hua Kwan Hospital, Singapore, Singapore; 6 Medical Services, St Luke's Hospital, Singapore, Singapore; 7 Medical Services, St Andrew's Community Hospital, Singapore, Singapore; 8 Bright Vision Hospital, Singapore, Singapore; 9 Department of Rehabilitation Medicine, Singapore General Hospital, Singapore, Singapore; 10 Genome Institute of Singapore, Agency for Science, Technology, and Research, Singapore, Singapore; 11 Graduate School for Integrative Science and Engineering, National University of Singapore, Singapore, Singapore; 12 Department of Statistics and Applied Probability, National University of Singapore, Singapore, Singapore; 13 Ministry of Health, Singapore, Singapore; Federal University of Rio de Janeiro, Brazil

## Abstract

**Objectives:**

The relationship between disability and comorbidity on mortality is widely perceived as additive in clinical models of frailty.

**Design:**

National data were retrospectively extracted from medical records of community hospital.

**Data Sources:**

There were of 12,804 acutely-disabled patients admitted for inpatient rehabilitation in Singapore rehabilitation community hospitals from 1996 through 2005 were followed up for death till 31 December 2011.

**Outcome Measure:**

Cox proportional-hazards regression to assess the interaction of comorbidity and disability at discharge on all-cause mortality.

**Results:**

During a median follow-up of 10.9 years, there were 8,565 deaths (66.9%). The mean age was 73.0 (standard deviation: 11.5) years. Independent risk factors of mortality were higher comorbidity (p<0.001), severity of disability at discharge (p<0.001), being widowed (adjusted hazard ratio [aHR]: 1.38, 95% confidence interval [CI]:1.25–1.53), low socioeconomic status (aHR:1.40, 95%CI:1.29–1.53), discharge to nursing home (aHR:1.14, 95%CI:1.05–1.22) and re-admission into acute care (aHR:1.54, 95%CI:1.45–1.65). In the main effects model, those with high comorbidity had an aHR = 2.41 (95%CI:2.13–2.72) whereas those with total disability had an aHR = 2.28 (95%CI:2.12–2.46). In the interaction model, synergistic interaction existed between comorbidity and disability (p<0.001) where those with high comorbidity and total disability had much higher aHR = 6.57 (95%CI:5.15–8.37).

**Conclusions:**

Patients with greater comorbidity and disability at discharge, discharge to nursing home or re-admission into acute care, lower socioeconomic status and being widowed had higher mortality risk. Our results identified predictive variables of mortality that map well onto the frailty cascade model. Increasing comorbidity and disability interacted synergistically to increase mortality risk.

## Introduction

Frailty is an important clinical syndrome that increases an individual's vulnerability to adverse health outcomes and mortality.[1]–[3] The classic frailty cascade model describes how age-related physiological deterioration, stressful events, functional decline, recovery, disability, hospitalization, and institutionalization ultimately contribute to premature death in older individuals.[4] Older patients admitted for acute illness may have multiple chronic conditions,[5] experience disability,[6] and become institutionalized following discharge from the acute care setting.[7] Development of physical frailty in older individuals is greatly impacted by age-related physiological deterioration and a wide range of diseases and medical conditions.[8], [9] Comorbidity is the concurrent presence of two or more medically diagnosed diseases,[3] and is commonly experienced by older individuals.[8]–[11] Comorbidity has also been shown to increase the risk of premature death in a variety of patients such as breast cancer,[12] essential hypertension or diabetes[13] and spinal cord injury.[14] Disability can be characterized as dependency in performing activities of daily living[3] and are common in older individuals. Elderly patients are particularly vulnerable to disability and functional decline after acute hospitalizations due to enforced immobilization and deconditioning.[3], [5], [15] Development of disabilities in older patients results in not only substantial costs, but also increased long term morbidity and mortality.[16]–[18] For example, hip fracture may reduce life expectancy by as much as 25%.[16]

Some authors have perceived comorbidity as an etiologic risk factor and disability as an outcome of frailty.[19], [20] Other models of frailty such as the frailty cascade,[4] Frailty Index (FI)[21], [22] and Frailty Scale (FS)[23], [24] include both disability and comorbidity as risk factors of premature mortality outcome.[25], [26] Although relationship between frailty and mortality has been shown previously,[27], [28] the relative and combined impact of specific aspects of frailty (e.g. comorbidity and disability) remain unclear. The specific aims of this study were to identify predictors of mortality and to describe the combined effect of comorbidity and disability on mortality with reference to models of frailty.

## Methods

### Study Population

The study population was a historical national cohort taken from a database of all patients admitted to all community hospitals in Singapore between 1996 and 2005. Community hospitals are rehabilitation hospitals that cater to patients who are fit for discharge from acute hospitals but require inpatient convalescent or subacute rehabilitative care before returning to a final domiciliary site.[29] We excluded patients who died during their hospital stay in the community hospital (n = 24). Inclusion criteria for this study were:

1. First admission to community hospitals for inpatient rehabilitation from acute disability (e.g. stroke and hip fractures); and
2. Disability assessed at discharge.

The study was approved by the National University of Singapore Institutional Review Board (NUS-IRB) and ethics committees of Ang Mo Kio Thye Hua Kwan Hospital, Bright Vision Hospital, St Andrew's Community Hospital and St Luke's Hospital. Written informed consent of the patient was waived by approving NUS-IRB. The corresponding author and all research nurses have taken the oath of confidentiality under Singapore's Official Secrets Act and only the minimum number of research personnel had access to the de-identified dataset.

### Data Extraction

Trained research nurses performed data extraction from non-computerized medical records between November 2005 and August 2008. Multiple iterations of data cleaning and verification were performed. An independent physician analyzed a 10% random sample of patients for data extraction accuracy and the error rate was 0.07%.

### Covariates

A number of covariates were controlled to elicit the specific effects of comorbidity and disability including socio-demographic variables such as age group (18–64, ≥65 years), gender (female, male), ethnicity (Chinese, Malay, Indian, others), marital status (single, married, widowed, separated or divorced), caregiver availability (no, yes) and length of hospital stay. Hospitalization subsidy level served as a proxy measure for socioeconomic status (SES). In Singapore, 81% of public hospitals' beds (B2 and C class) are highly subsidized.[29] B2+ and B1 class lower subsidy and A class have no subsidy. For this study, SES was classified into three groups: high SES (A, B1 and B2+ class), moderate SES (B2 class) and low SES (C class). Discharge destinations of patients were home (n = 9,774), acute hospital (n = 1,583), nursing home (n = 1,251) and others (n = 196). As the numbers who were discharged to other destinations were low, they were excluded from multivariate models.

### Exposures of interest

#### Comorbidity

Comorbidity was measured using the Charlson comorbidity index (CCI).[12] Comorbidity data was manually extracted by trained nurses by reviewing the patients' medical problem list and complete medical records. The CCI measures the burden of medical illnesses and comprises of 19 different categories. The overall CCI score is a summation of weighted scores. A higher score reflects greater cumulative disease burden. We categorized index scores into four groups: no comorbidity (0), low comorbidity (1–3), moderate comorbidity (4–6) and high comorbidity (7–16). The Charlson comorbidity score also has been shown to have a high inter-rater reliability of a kappa score of 0.93.[30]

#### Disability at discharge

As recommended by the Singapore Ministry of Health guidelines, disability was assessed in both community hospitals at admission and discharge[29] using the Shah modified Barthel Index (BI). This is usually done by qualified physiotherapists and/or occupational therapists in all community hospitals. The BI is a reliable and widely-used assessment for functional status[31] and disability.[32], [33] It comprises of 10 items measuring distinct activities of daily living (ADL). The BI utilizes a five-point scale for each ADL and has a maximum score of 100.[34] BI scores are categorized by the severity of disability: 0–24 (total disability), 25–49 (severe disability), 50–74 (moderate disability), 75–90 (mild disability), 91–99 (minimal disability) and 100 (no disability).[34] For this study, the last three categories were collapsed into one category (75–100) and termed “no or mild disability” because of small sample sizes. The reliability of the BI has been demonstrated in numerous studies and test-retest, intra-rater and inter-rater reliability have been shown to be high by correlation methods (r = 0.87, 0.71–0.99 and 0.75–0.99 respectively).[35], [36] We chose to use discharge BI score of patients admitted for rehabilitation because this represented the best functional status after optimization with therapy.

### Determination of Endpoint

The primary endpoint was mortality. Time-to-event was calculated from the time of discharge disability assessment to the time of death. Subjects who remained alive at study closure were censored at 31 December 2011. Survival status was obtained from national death databases.

### Statistical Analyses

For bivariate analyses, Chi-square tests were performed for categorical variables, log rank test for survival time, Kruskal-Wallis tests for length of stay.

Kaplan–Meier overall survival curves were compared for exposures of interest, namely comorbidity and disability, using two-sided log rank tests. The Cox proportional-hazards regression was implemented to identify predictors of mortality within the cohort. Schoenfeld residuals were used to test the proportional-hazards (PH) assumption after model fitting. In the event of violation of PH assumption for a specific covariate, the same covariate was included as a stratifying factor in the Cox model. Likelihood ratio tests were used to compare nested models to identify significant predictors of mortality. In the synergistic model, the multiplicative interaction term of comorbidity and disability was included in the model. Potential confounders were adjusted in multiplicative interaction model. All analyses were performed using STATA version 11 (StataCorp LP, USA) with the significance level set at 0.05.

## Results

19,360 community hospital patient admissions were initially considered. 2,314 (12.0%) non-rehabilitation were first excluded, followed by 2,604 (15.3%) patients with second or subsequent admissions. A total of 14,442 first rehabilitation admission patients were thus recruited. Among them, 1,638 patients (11.3%) had missing discharge disability status. The final cohort consisted of 12,804 patients (**Figure S1**), of whom 4,239 (33.1%) were alive at the end of the study. The median follow-up time across the cohort was 10.9 years (interquartile range [IQR]: 8.5–13.3 years). The mean age of the cohort at admission was 73.0 (standard deviation [SD]: 11.5) years, mean discharge BI scores was 60.4 (SD = 29.0), median length of stay was 32.0 days (IQR: 20.0–47.0), and median Charlson comorbidity index score was 3 (IQR: 1–5). Patients who had total disability were more likely to be admitted for stroke (p<0.001), moderate SES (p<0.001) and discharged to acute hospital (25.4%) compared to other disability groups. Length of stay was not significantly different across the four disability groups (p = 0.053) (**Table 1**).

**Table 1:** Social demographics by discharge disability.

	Total (n = 12,804)	No or mild disability (n = 5082)	Moderate disability (n = 3750)	Severe disability (n = 1886)	Total disability (n = 2086)	P-value^a^
Year of admission						
1996	585 (4.6)	116 (2.3)	100 (2.7)	60 (3.2)	309 (14.8)	<0.001
1997	1291 (10.1)	348 (6.9)	384 (10.2)	196 (10.4)	363 (17.4)	
1998	1316 (10.3)	382 (7.5)	409 (10.9)	245 (13.0)	280 (13.4)	
1999	1357 (10.6)	571 (11.2)	429 (11.4)	196 (10.4)	161 (7.7)	
2000	1205 (9.4)	519 (10.2)	374 (10.0)	175 (9.3)	137 (6.6)	
2001	1239 (9.7)	512 (10.1)	371 (9.9)	194 (10.3)	162 (7.8)	
2002	1113 (8.7)	462 (9.1)	365 (9.7)	141 (7.5)	145 (7.0)	
2003	1368 (10.7)	552 (10.9)	379 (10.1)	224 (11.9)	213 (10.2)	
2004	1631 (12.7)	800 (15.7)	450 (12.0)	228 (12.1)	153 (7.3)	
2005	1699 (13.3)	820 (16.1)	489 (13.0)	227 (12.0)	163 (7.8)	
Primary diagnosis at admission						
Stroke	5240 (40.9)	1557 (30.6)	1536 (41.0)	880 (46.7)	1267 (60.7)	<0.001
Fracture	3781 (29.5)	1733 (34.1)	1188 (31.7)	464 (24.6)	396 (19.0)	
Amputation	299 (2.3)	135 (2.7)	98 (2.6)	29 (1.5)	37 (1.8)	
Others	2434 (19.0)	1110 (21.8)	663 (17.7)	375 (19.9)	286 (13.7)	
Lower limb arthroplasty	362 (2.8)	257 (5.1)	75 (2.0)	20 (1.1)	10 (0.5)	
Cancer	243 (1.9)	113 (2.2)	66 (1.8)	39 (2.1)	25 (1.2)	
Falls	232 (1.8)	104 (2.1)	77 (2.1)	31 (1.6)	20 (1.0)	
Pneumonia	213 (1.7)	73 (1.4)	47 (1.3)	48 (2.6)	45 (2.2)	
Gender						
Female	7463 (58.3)	2934 (57.7)	2302 (61.4)	1057 (56.0)	1170 (56.1)	<0.001
Male	5341 (41.7)	2148 (42.3)	1448 (38.6)	829 (44.0)	916 (43.9)	
Age group						
Age: 18–64 years	2671 (20.9)	1359 (26.7)	667 (17.8)	289 (15.3)	356 (17.1)	<0.001
Age: ≥65 years	10133 (79.1)	3723 (73.3)	3083 (82.2)	1597 (84.7)	1730 (82.9)	
Length of stay (days), median (25^th^–75^th^)	32 (20–47)	32 (22–47)	33 (23–47)	33 (21–46)	33 (19–49)	0.053
Community hospital						
A	3240 (25.3)	1750 (34.4)	755 (20.1)	416 (22.1)	319 (15.3)	<0.001
B	6727 (52.5)	2591 (51)	2349 (62.6)	1115 (59.1)	672 (32.2)	
C	2381 (18.6)	578 (11.4)	513 (13.7)	301 (16.0)	989 (47.4)	
D	456 (3.6)	163 (3.2)	133 (3.6)	54 (2.9)	106 (5.1)	
Ethnicity						
Chinese	11293 (88.2)	4578 (90.1)	3315 (88.4)	1617 (85.7)	1783 (85.5)	<0.001
Malay	848 (6.6)	234 (4.6)	264 (7.0)	167 (8.9)	183 (8.8)	
Indian	518 (4.1)	206 (4.1)	134 (3.6)	82 (4.4)	96 (4.6)	
Others	145 (1.1)	64 (1.3)	37 (1.0)	20 (1.1)	24 (1.2)	
Marital status						
Single	1099 (8.6)	675 (13.3)	232 (6.2)	86 (4.6)	106 (5.1)	<0.001
Married	5483 (42.8)	2080 (40.9)	1594 (42.5)	849 (45.0)	960 (46.0)	
Widowed	5853 (45.7)	2134 (42)	1826 (48.7)	917 (48.6)	976 (46.8)	
Separated/Divorced	369 (2.9)	193 (3.8)	98 (2.6)	34 (1.8)	44 (2.1)	
Caregiver						
No	1257 (9.8)	744 (14.6)	294 (7.8)	110 (5.8)	109 (5.2)	<0.001
Yes	11547 (90.2)	4338 (85.4)	3456 (92.2)	1776 (94.2)	1977 (94.8)	
Socioeconomic status						
High	1207 (9.4)	424 (8.3)	445 (11.9)	178 (9.4)	160 (7.7)	<0.001
Moderate	4478 (35.0)	1480 (29.1)	1242 (33.1)	630 (33.4)	1126 (54.0)	
Low	7119 (55.6)	3178 (62.5)	2063 (55.0)	1078 (57.2)	800 (38.4)	
Comorbidity burden (Charlson comorbidity index)						
None (0)	2377 (18.6)	1344 (26.5)	652 (17.4)	196 (10.4)	185 (8.9)	<0.001
Low (1–3)	5878 (45.9)	2348 (46.2)	1742 (46.5)	864 (45.8)	924 (44.3)	
Moderate (4–6)	4012 (31.3)	1217 (24)	1202 (32.1)	713 (37.8)	880 (42.2)	
High (≥7)	537 (4.2)	173 (3.4)	154 (4.1)	113 (6.0)	97 (4.7)	
Discharge destination						
Home	9774 (77.5)	4466 (88.8)	2844 (77.3)	1215 (65.6)	1249 (61.1)	<0.001
Nursing Home	1251 (9.9)	310 (6.2)	397 (10.8)	267 (14.4)	277 (13.6)	
Acute hospital	1583 (12.6)	255 (5.1)	440 (12.0)	369 (19.9)	519 (25.4)	

a P-value: Chi-square test for categorical variables and Kruskal-Wallis test for length of stay.

### Bivariate analyses (Table 2)

**Figure 1:**
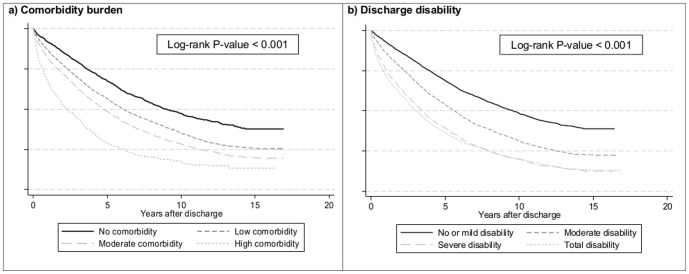
Kaplan-Meier survival curve by comorbidity burden, discharge disability and discharge destination.

Among those who died, significantly more were male (p<0.001), older (≥65 years, p<0.001), widowed (p<0.001), had a caregiver (p<0.001), from low or moderate SES group (p<0.001), had greater comorbidity burden (p<0.001) and had greater disability (p<0.001) at discharge, and shorter length of stay (p = 0.005) (**Table 2** **,** **Figure 1**). Tests on proportional hazards assumption were performed. Gender, age group (18–64 vs. ≥65 years), year of admission and primary diagnosis at admission violated the proportional hazards assumption. Thus, we stratified the study sample by these variables and obtained pooled estimates. Patients with low, moderate or high comorbidity had 1.43 (95% confidence interval (CI): 1.34–1.54, p<0.001), 1.94 (95% CI: 1.79–2.09, p<0.001) and 2.80 (95% CI: 2.49–3.16, p<0.001) times mortality risks respectively compared to patients with no comorbidity. Patients with moderate, severe and total disability at discharge had 1.54 (95% CI: 1.46–1.63, p<0.001), 2.27 (95% CI: 2.12–2.42, p<0.001) and 2.44 (95% CI: 2.28–2.61, p<0.001) times mortality risks respectively compared to patients with no or mild disability.

**Table 2:** Social demographics by death status at time of censoring and bivariate model of all-cause mortality for hazard ratio.

	Alive	Dead	Hazard ratio (95% Confidence Interval)	P-value^a^
	(n = 4239)	(n = 8565)		
Year of admission			Stratification	variable
1996	112 (2.6)	473 (5.5)		
1997	266 (6.3)	1025 (12.0)		
1998	270 (6.4)	1046 (12.2)		
1999	355 (8.4)	1002 (11.7)		
2000	349 (8.2)	856 (10.0)		
2001	384 (9.1)	855 (10.0)		
2002	365 (8.6)	748 (8.7)		
2003	534 (12.6)	834 (9.7)		
2004	726 (17.1)	905 (10.6)		
2005	878 (20.7)	821 (9.6)		
Primary diagnosis at admission			Stratification	variable
Stroke	1708 (40.3)	3532 (41.2)		
Fracture	1343 (31.7)	2438 (28.5)		
Amputation	72 (1.7)	227 (2.7)		
Others	745 (17.6)	1689 (19.7)		
Lower limb arthroplasty	237 (5.6)	125 (1.5)		
Cancer	47 (1.1)	196 (2.3)		
Falls	46 (1.1)	186 (2.2)		
Pneumonia	41 (1.0)	172 (2.0)		
Gender			Stratification	variable
Female	2619 (61.8)	4844 (56.6)		
Male	1620 (38.2)	3721 (43.4)		
Age group			Stratification	variable
Age: 18–64 years	1502 (35.4)	1169 (13.7)		
Age: ≥65 years	2737 (64.6)	7396 (86.4)		
Length of stay (days), median (25^th^–75^th^)	31 (20–45)	31 (20–47)	0.999 (0.998–1.000)	0.010
Community hospital				
A	1164 (27.5)	2076 (24.2)	1.00 (ref)	
B	2159 (50.9)	4568 (53.3)	1.04 (0.99–1.10)	0.126
C	744 (17.6)	1637 (19.1)	1.06 (0.99–1.14)	0.084
D	172 (4.1)	284 (3.3)	1.43 (1.25–1.63)	<0.001
Ethnicity				
Chinese	3738 (88.2)	7555 (88.2)	1.00 (ref)	
Malay	262 (6.2)	586 (6.8)	1.23 (1.13–1.35)	<0.001
Indian	186 (4.4)	332 (3.9)	1.00 (0.90–1.13)	0.933
Others	53 (1.3)	92 (1.1)	0.89 (0.72–1.10)	0.269
Marital status				
Single	518 (12.2)	581 (6.8)	1.00 (ref)	
Married	1939 (45.7)	3544 (41.4)	1.19 (1.08–1.30)	<0.001
Widowed	1615 (38.1)	4238 (49.5)	1.47 (1.34–1.61)	<0.001
Separated/Divorced	167 (3.9)	202 (2.4)	1.10 (0.94–1.30)	0.237
Caregiver				
No	485 (11.4)	772 (9.0)	1.00 (ref)	
Yes	3754 (88.6)	7793 (91.0)	1.19 (1.10–1.28)	<0.001
Socioeconomic status				
High	424 (10.0)	783 (9.1)	1.00 (ref)	
Moderate	1361 (32.1)	3117 (36.4)	1.12 (1.04–1.22)	0.005
Low	2454 (57.9)	4665 (54.5)	1.31 (1.21–1.42)	<0.001
Comorbidity burden (Charlson comorbidity index)				
None (0)	1107 (26.1)	1270 (14.8)	1.00 (ref)	
Low (1–3)	1992 (47.0)	3886 (45.4)	1.43 (1.34–1.54)	<0.001
Moderate (4–6)	1049 (24.8)	2963 (34.6)	1.94 (1.79–2.09)	<0.001
High (≥7)	91 (2.2)	446 (5.2)	2.80 (2.49–3.16)	<0.001
Discharge disability measured by Barthel Index				
No or mild disability (75–100)	2448 (57.8)	2634 (30.8)	1.00 (ref)	
Moderate disability (50–74)	1108 (26.1)	2642 (30.9)	1.54 (1.46–1.63)	<0.001
Severe disability (25–49)	349 (8.2)	1537 (18.0)	2.27 (2.12–2.42)	<0.001
Total disability (0–24)	334 (7.9)	1752 (20.5)	2.44 (2.28–2.61)	<0.001
Discharge destination				
Home	3579 (86.0)	6195 (73.4)	1.00 (ref)	
Nursing Home	291 (7.0)	960 (11.4)	1.37 (1.28–1.47)	<0.001
Acute hospital	293 (7.0)	1290 (15.3)	1.96 (1.84–2.09)	<0.001

a P-value: Cox-proportional hazard model: stratified by age group (18–64, 65 and above), year of admission, gender, primary diagnosis at admission (stroke, fracture, amputation, lower limb arthroplasty, falls, others).

### Multivariate analyses

Length of stay was dropped from final model as it became insignificant after adjusting for discharge destination (p = 0.662). Significant predictors of mortality in the final multivariate model were discharge destination, SES group, community hospital and marital status (**Table 3**). Patients discharged to nursing homes and acute hospitals had 1.14 (95% CI: 1.05–1.22, p = 0.001) and 1.54 (95% CI: 1.45–1.65, p<0.001) times mortality risk respectively compared to those discharged to home. Patients from moderate or low SES group had 1.12 (95% CI: 1.03–1.22, p = 0.007) and 1.40 (95% CI: 1.29–1.53, p<0.001) times greater mortality risk respectively compared to those in high SES group. Patients who were widowed had 1.38 (95% CI: 1.25–1.53, p<0.001) times mortality risk compared to singles.

**Table 3:** Multivariate model of all-cause mortality in patients admitted to Singapore community hospitals from 1996 to 2005.

		Hazard ratio (95% Confidence Interval)	P-value^a^
**Comorbidity X**	**Disability**		
No	No or mild	1.00 (ref)	
No	Moderate	1.77 (1.55–2.01)	<0.001
No	Severe	3.09 (2.58–3.71)	<0.001
No	Total	2.46 (2.01–3.01)	<0.001
Low	No or mild	1.47 (1.33–1.64)	<0.001
Low	Moderate	2.03 (1.82–2.26)	<0.001
Low	Severe	3.07 (2.72–3.46)	<0.001
Low	Total	3.22 (2.84–3.65)	<0.001
Moderate	No or mild	1.99 (1.76–2.24)	<0.001
Moderate	Moderate	2.69 (2.39–3.03)	<0.001
Moderate	Severe	3.29 (2.89–3.74)	<0.001
Moderate	Total	4.42 (3.89–5.03)	<0.001
High	No or mild	3.09 (2.51–3.81)	<0.001
High	Moderate	3.64 (2.96–4.46)	<0.001
High	Severe	5.03 (3.98–6.35)	<0.001
High	Total	6.57 (5.15–8.37)	<0.001
Discharge destination			
Home		1.00 (ref)	
Nursing Home		1.14 (1.05–1.22)	0.001
Acute hospital		1.54 (1.45–1.65)	<0.001
Socioeconomic status			
High group		1.00 (ref)	
Moderate group		1.12 (1.03–1.22)	0.007
Low group		1.40 (1.29–1.53)	<0.001
Community hospital			
A		1.00 (ref)	
B		1.10 (1.04–1.17)	0.001
C		0.94 (0.87–1.02)	0.055
D		1.21 (1.05–1.38)	0.007
Marital status			
Single		1.00 (ref)	
Married		1.14 (1.03–1.26)	0.009
Widowed		1.38 (1.25–1.53)	<0.001
Separated/Divorced		1.12 (0.95–1.32)	0.190

a Cox-proportional hazard model: stratified by age group (18–64, 65 and above), year of admission (1996 to 2005), gender (female, male), primary diagnosis at admission (stroke, fracture, amputation, lower limb arthroplasty, falls, others).

**Figure 2:**
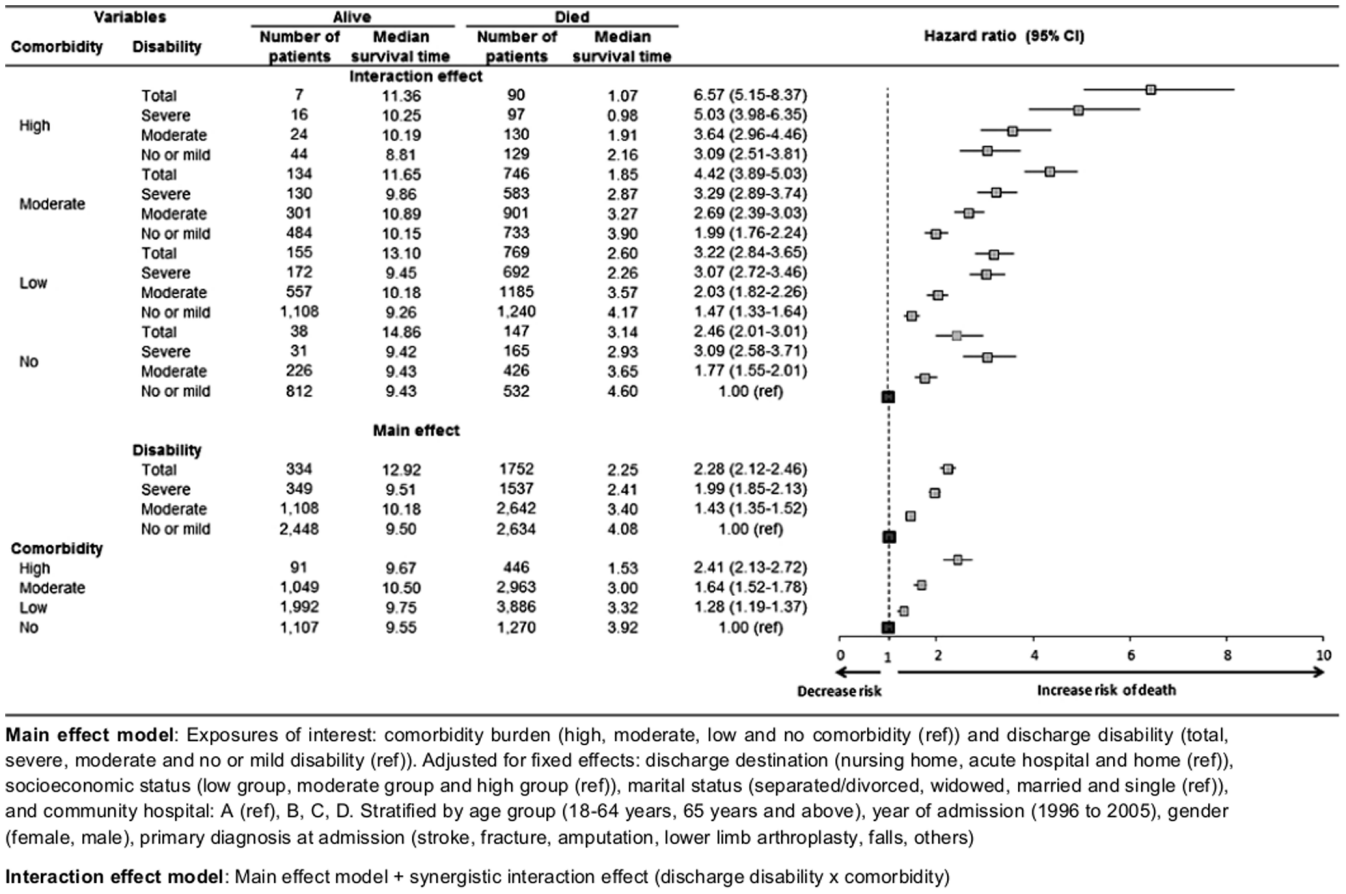
Multiplicative interaction effect of comorbidity and disability in patients admitted to Singapore community hospitals from 1996 to 2005.

The interaction between comorbidity and disability at discharge was significant in the final synergistic interaction model of mortality risk (p<0.001) (**Figure 2** **, Figure S2**). Patients with highest mortality risks had both comorbidity and disability compared to patients with either comorbidity or disability alone (**Figure 2**). Among patients who had high comorbidity, patients with no or mild, moderate, severe and total disability had 3.09 (95% CI: 2.51–3.81, p<0.001), 3.64 (95% CI: 2.96–4.46, p<0.001), 5.03 (95% CI: 3.98–6.35, p<0.001) and 6.57 (95% CI: 5.15–8.37, p<0.001) times greater mortality risk respectively compared to those with no comorbidity and no or mild disability (**Table 3** **,** **Figure 2**).

## Discussion

A national cohort of patients admitted for step-down inpatient rehabilitation after acute hospitalization was followed-up for up to 16 years. Comorbidity, discharge disability, widowhood, low socioeconomic status, readmission into acute care and institutionalization were independent predictors of mortality, reflecting the main elements in models of frailty.[20]–[24] Our results also demonstrated a novel synergistic effect between comorbidity and discharge disability on long-term mortality.[23], [24]

Our results identified predictive variables of mortality that map well onto the frailty cascade model and quantified the increased risks of each of these factors.[4] For example, in the presence of physiological decline (age-related) across multiple physiological systems aggravated by disease processes (comorbidity), an acute physical event (hospitalization) may cause a negative cascade of events from disability, healthcare utilization (readmission and institutionalization) to eventually death. One other integrated conceptual model of frailty postulated that life course determinants and diseases may lead to frailty and adverse outcomes.[37]–[39] This model of frailty can also be supported by our findings. For example, widowhood, low socioeconomic status (life course determinants) and comorbidity (diseases) result in disability (physical frailty), institutionalization and death (adverse outcomes). Hence, our results statistically validate earlier theoretical models identifying factors contributing to mortality in frail individuals.

Current models of clinical frailty that combine disability and comorbidity on an additive scale may underestimate the mortality risk, especially in the high-risk groups. The interaction between comorbidity and disability is better considered as a “comorbidity-disability complex”. This complex is a central component of many conceptual models of frailty.[3], [4], [17], [40] However, in contrast with current models that assume an additive effect of comorbidity and disability on mortality risk, our findings provide evidence that the effect of this comorbidity-disability complex on mortality risk is synergistic. To our knowledge, no previous study has reported a combined synergistic effect of comorbidity and disability on long-term mortality risk. One approach to understanding the synergistic interaction effect is to tease out how comorbidity and disability impact each other. For example, it can be conceived that comorbidities increase the likelihood of hospitalizations, leading to disability, decreased mobility, sarcopenia, a higher level of frailty, and potentially further comorbidities. These factors are likely to have bidirectional influences and may continuously propagate in a vicious cycle that perpetuates itself until physical frailty results in premature death.

Clinical management of disability, comorbidity and frailty each has its unique challenges. Disabled older patients are at greater risk of social isolation, institutionalization, and new chronic diseases and initiation of frailty and initially of frailty.[3] Hence, medical care for the disabled involves rehabilitation to maximize function and prevent further decline. Fragmented sub-specialized care focused on single disease leads to complications in patients with multiple comorbidities due to complex relationships between conditions and their treatments.[41] Frail patients also have additional needs beyond those of underlying comorbidity and disability as they are vulnerable to other stressors such as hospitalization, under-nutrition and falls. Additional care is therefore needed to treat pathologic causes of progressive weakness, prevent iatrogenesis and reduce risk factors that result in disability.[3]

### Strengths and limitations

The strengths of our study are a large study sample with long follow-up of up to 16 years. These provided the opportunity to elicit complex long-term relationships such as the synergistic interaction between comorbidity and disability on mortality. The database tracked all patients admitted to community hospitals during the study period and hence was a nationally representative study population. The limitation of this study is the use of retrospective data and incompleteness of records which could have introduced data entry biases. Furthermore, the study was limited to investigating variables that the database included and did not consider other factors may also affect long-term outcomes (e.g. cognition, mental health, quality of life and healthcare decision-making). In addition, we excluded 24 patients who died during their hospital stay. As these patients had poorer admission functional scores (mean = 23.9 vs. 46.3) and greater comorbidity burden (mean = 4.5 vs. 2.9) compared to patients in our study, our current risk estimates would be slightly conservative. Finally, the study was completed in an Asian population within a developed economy and an advanced healthcare system. Generalization of these findings to Western and other populations should be done with care.

## Conclusions

This study confirmed that comorbidity and disability are independent predictors of mortality risks in patients after discharge from acute hospitalizations. In addition to widowhood and institutionalization, we also found a novel synergistic interaction effect of the comorbidity-disability complex independent on mortality risk. Future research should consider the feasibility and value of replicating this prospective study in non-Asian populations. The mechanisms through which the comorbidity-disability complex impacts mortality also warrants further investigation.

## Supporting Information

Figure S1 **Flowchart of selection criteria.**
(TIF)

Figure S2 **Kaplan-Meier survival curves stratified by comorbidity and discharge disability.**
(TIF)
