# Supplementary figures and images for: The Synergistic Effect of Functional Status and Comorbidity Burden on Mortality: A 16-Year Survival Analysis
Source: PLoS One. 2014 Aug 29;9(8):e106248. doi: 10.1371/journal.pone.0106248 (PMC4149557; doi:10.1371/journal.pone.0106248)

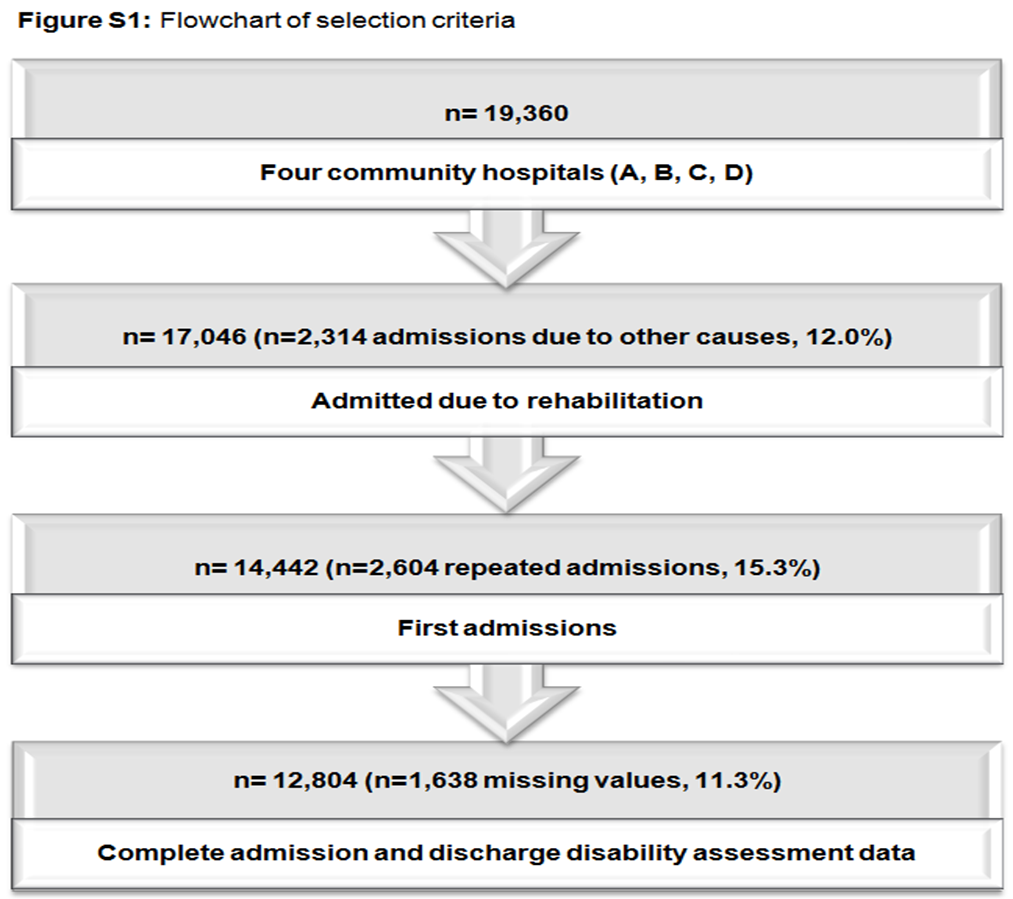

Supplement: Figure S1 — Flowchart of selection criteria. (TIF) [file pone.0106248.s001.tif]

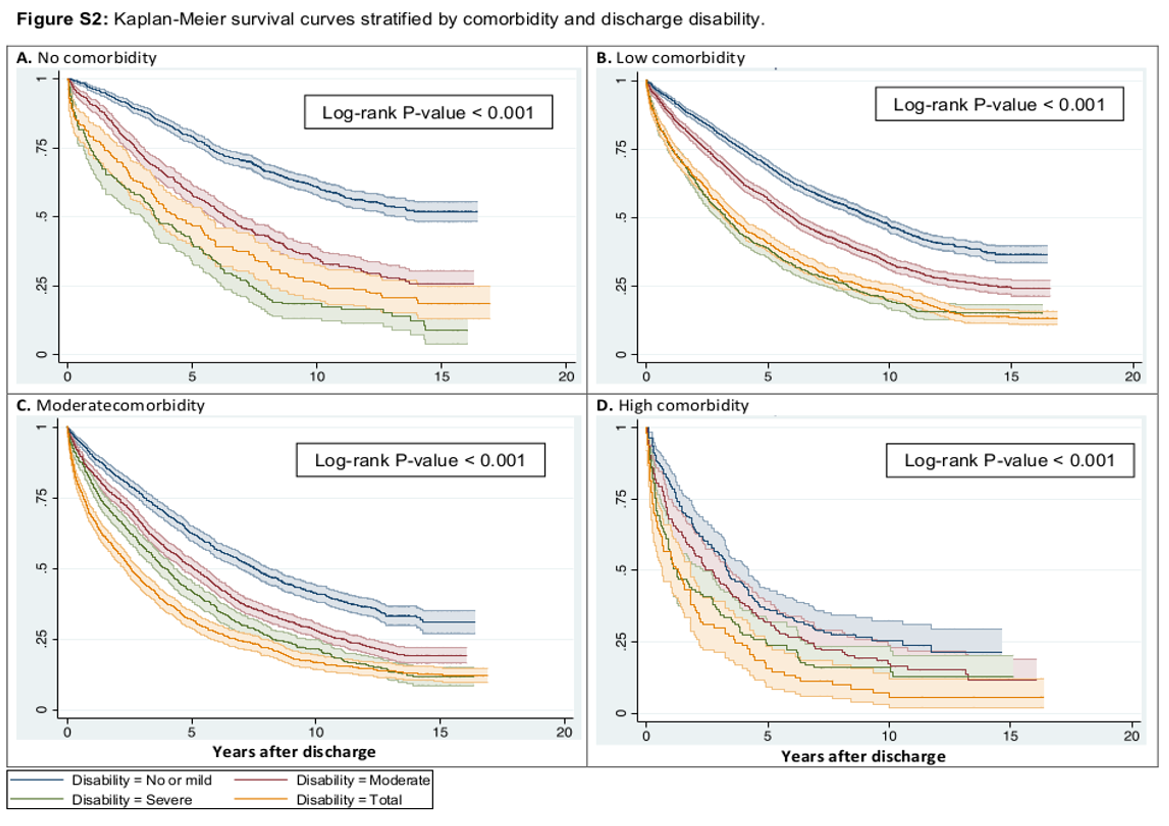

Supplement: Figure S2 — Kaplan-Meier survival curves stratified by comorbidity and discharge disability. (TIF) [file pone.0106248.s002.tif]
